# Eruptive Angiomatosis Triggered by COVID-19 Vaccination

**DOI:** 10.7759/cureus.22907

**Published:** 2022-03-07

**Authors:** Mohammed Shanshal

**Affiliations:** 1 Dermatology, Basildon University Hospital, Basildon, GBR

**Keywords:** benign vascular lesions, eruptive angiomatosis, covid-19, covid-19 vaccine adverse effects, covid-19 vaccination

## Abstract

Despite meeting strict standards of quality, safety, and effectiveness, rare systemic and cutaneous side effects of coronavirus disease 2019 (COVID-19) vaccinations continue to be reported throughout the world. We report a case of eruptive cherry angiomatosis in a female following her first dose of COVID-19 vaccination with subsequent crops appearing after the second dose. The biopsy revealed dilated capillaries within the superficial dermis consistent with the clinical diagnosis.

## Introduction

Since the World Health Organization declared it a global pandemic in March 2020, coronavirus disease 2019 (COVID-19), caused by severe acute respiratory syndrome coronavirus 2 (SARS-CoV-2), has been spreading all across the world. In an effort to control its spread and associated fatality rates, COVID-19 has spurred the biggest vaccination campaign in history. Despite the well-documented safety profile of these vaccines, rare incidences of cutaneous and systemic adverse effects have been reported as millions of people receive their vaccinations worldwide [1,2].

## Case presentation

A 55-year-old female presented to the Ambulatory Care Unit at the Basildon University Hospital with a rash affecting her chest area as well as the abdominal wall, breast, and arms. She had first noticed these lesions developing in February 2021, 10 days after receiving her first dose of the Oxford-AstraZeneca COVID-19 vaccine. The rash had consisted of multiple red-purplish papules associated with flu-like illness. She had subsequently noticed more crops of these lesions appear after she had received her second dose in the middle of April. The lesions were asymptomatic and had not faded. The patient had been under the care of the Rheumatology team for psoriatic arthritis. She was on sulfasalazine and Enbrel injection. Her skin was normal and she only experienced nail changes associated with her psoriasis.

**Figure 1:**
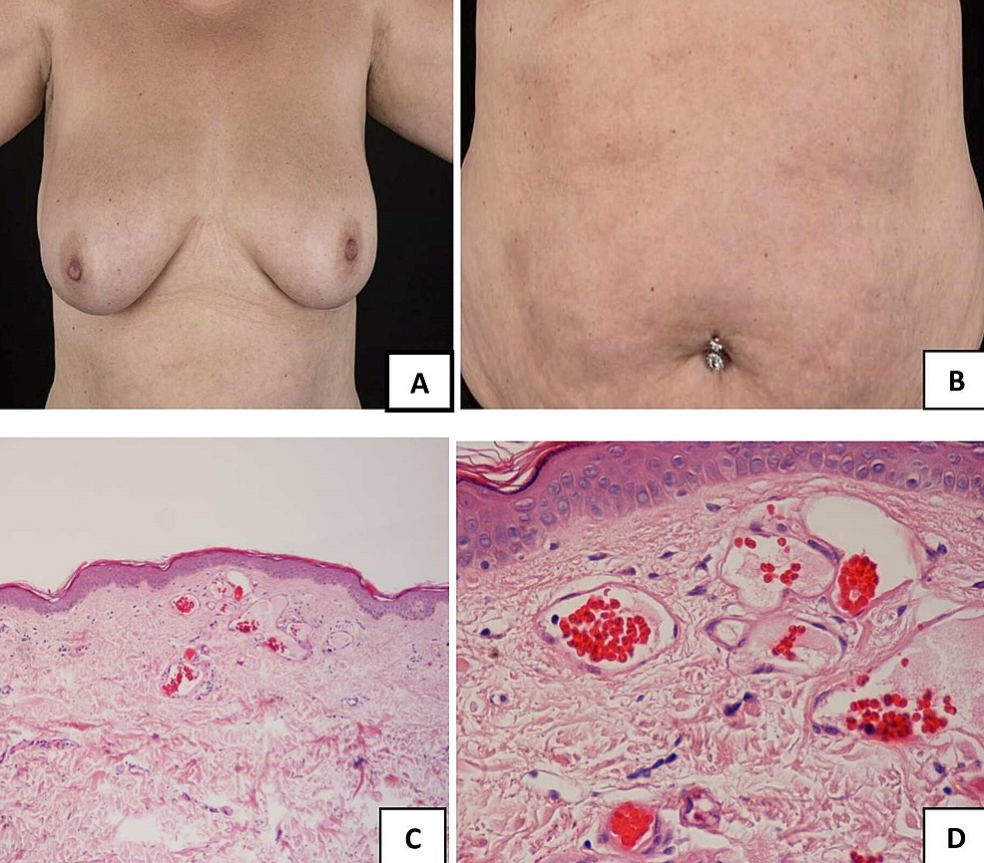
COVID-19-triggered cherry angiomatosis A, B: erythematous papules involving the chest, breasts, and abdomen; C, D: hematoxylin and eosin stain (20x and 40x) showing dilated interconnecting capillaries within the superficial dermis COVID-19: coronavirus disease 2019

On examination, multiple erythematous papules of 2-3 mm were detected predominantly on her chest and abdominal wall; scattered papules on her arms and legs were also observed. Her blood exam on admission showed normal liver function, full blood count, clotting screen, renal function, and a C-reactive protein of 1 mg/L. She had a low positive antinuclear antibody level; however, this was of uncertain significance since she had normal myeloperoxidase and low proteinase 3 antibodies. Punch biopsy of the upper abdomen revealed dilated interconnecting capillaries within the superficial dermis consistent with the clinical picture of cherry angioma (Figures 1A-1D).

## Discussion

Based on the clinical appearance, the histopathological findings, and the temporal relationship between the first dose of vaccination and the flare-up following the second dose, a diagnosis of eruptive angiomatosis triggered by COVID-19 vaccination was made. Cherry angioma is a benign vascular lesion of unknown etiology more commonly associated with old age, pregnancy, and somatic mutations in GNAQ and GNA11 genes [3]. Its eruptive form may be seen in association with lymphoproliferative disorders, certain medications, and the human herpesvirus-8 [4].

Vascular lesions including eruptive cherry angiomas have been reported secondary to COVID-19, which could be mediated by angiotensin-converting enzyme 2 (ACE2) receptors; subsequent endothelial damage has also been reported [5,6]. Mohta et al. have reported 12 cases of eruptive pseudoangiomatosis, a similar vascular lesion that commonly occurs in the pediatric age group and is associated with ChAdOx1 nCoV‐19 coronavirus vaccine (recombinant) in India [7]. The possible vascular injury [8,9] or immune dysregulation [10] caused by the COVID-19 vaccination could play a role in the development of these eruptive vascular lesions.

## Conclusions

Cherry angiomas, also known as Campbell de Morgan spots, are harmless and generally asymptomatic vascular lesions comprising dilated capillaries. They typically present as bright red or purplish papules of varying sizes, and they are more common in older adults and pregnant women. Eruptive cherry angiomas can occur in association with certain medications, viral infections, and lymphoproliferative disorders. We reported a case of new-onset eruptive angiomatosis following the COVID-19 vaccination in a female patient. The report highlights one of the cutaneous side effects of COVID-19 vaccines. Even though these rare adverse events are relevant, they should not undervalue the importance of immunization against COVID-19 and its overall safety profile.
